# Complexity of schistosome vector bulinine snails in Kenya: Insights from nuclear genome size variation, complete mitochondrial genome sequence, and morphometric analysis

**DOI:** 10.1371/journal.pntd.0013305

**Published:** 2025-07-14

**Authors:** Si-Ming Zhang, Coen M. Adema, Mohamed R. Habib, Abdelmalek Lekired, Marijan Posavi, Martina R. Laidemitt, Geoffrey M. Maina, Ibrahim N. Mwangi, Joseph M. Kinuthia, Martin W. Mutuku, Eric S. Loker

**Affiliations:** 1 Center for Evolutionary and Theoretical Immunology, Department of Biology, University of New Mexico, Albuquerque, New Mexico, United States of America; 2 Medical Malacology Department, Theodor Bilharz Research Institute, Giza, Egypt; 3 Centre for Biotechnology Research and Development, Kenya Medical Research Institute, Nairobi, Kenya; Natural History Museum, UNITED KINGDOM OF GREAT BRITAIN AND NORTHERN IRELAND

## Abstract

Investigations of nuclear genome size, complete mitochondrial genome (mitogenome) sequence, and morphometrics were conducted on specimens of *Bulinus* snails (Gastropoda: Planorbidae) collected from 14 locations across the east coast, central Kenya, and western Kenya around the Lake Victoria region (November 2013 and January 2024). Flow cytometry measurements of DNA content (C-value) revealed unexpected variation in nuclear genome size, with diploid *Bulinus africanus* and *B. forskalii* species groups showing C-values ranging from 0.76 to 1.98 pg, while tetraploid *B. truncatus* had a C-value of 1.82 pg. Additionally, C-values for six *B. globosus* specimens from different localities ranged from 1.43 to 1.98 pg. These findings suggest that bulinine snails, particularly the *B. africanus* species group, have undergone genome expansion, whole genome duplication (polyploidization), or both, which have not been previously recognized. Next-generation sequencing was performed to determine and annotate 14 complete mitogenome sequences. Despite the well-conserved arrangement of protein-coding genes, two versions of mtDNA genome structure, distinguished by the tRNA-D (Asp) location, were found, designated as DCF (Asp-Cys-Phe) type (in the *B. forskalii* group and the *B. truncatus*/*tropicus* complex) and CF (Cys-Phe) type (in the *B. africanus* group). Phylogenetic analyses based on complete mtDNA sequences of bulinines from Kenya, along with *cytochrome c oxidase subunit I* (*COX1*) sequences from various localities across Africa, contributed to resolving species identities and provided further support for the presence of multiple or cryptic species in the taxon *B. globosus*. A landmark-based morphometric analysis was ineffective in distinguishing these species. This study reveals unexpected nuclear genome size variation, provides new mitogenome sequences, and highlights the limitations of morphological analysis. It offers valuable insights into the cytogenetics, polyploidy, genomics, taxonomy, and evolution of bulinines, which serve as intermediate hosts for schistosomes responsible for human urogenital schistosomiasis and intestinal schistosomiasis in domestic and wild mammals.

## Author summary

Freshwater snails of the genus *Bulinus* play a crucial role in supporting the larval development of schistosomes and other trematode parasites responsible for significant diseases in humans and animals in Africa; yet knowledge of their genetics and genomics remains limited. Considerable contradictions exist regarding species identification and classification. Focusing on bulinine specimens from Kenya, we employed flow cytometry, next-generation sequencing, and morphometrics for a simultaneous investigation, making the data comparable and informative. This study uncovers unexpected genome size variation, suggests the presence of unrecognized genome expansion and/or whole genome duplication (polyploidization), provides new mitogenome data, clarifies phylogenetic relationships, illuminates genome evolution, highlights the limitations of morphological analysis, and raises questions about the cytogenetics and classification of the genus *Bulinus*.

## Introduction

Freshwater gastropod snails of the genus *Bulinus* Müller (1781) (family: Planorbidae) are the most medically important molluscan species in Africa [1]. These snails play an essential role in transmitting parasites that cause disease in humans and animals, serving as obligate intermediate hosts for the trematode parasite *Schistosoma haematobium*, the causative agent of urogenital schistosomiasis. Urogenital schistosomiasis accounts for over two-thirds of all schistosomiasis cases in Africa, which is home to approximately 90% of global cases [2–5]. In addition to *S. haematobium, Bulinus* snails also transmits other schistosomes, including *S. intercalatum* and *S. guineensis*, which cause intestinal schistosomiasis in humans, although they are less prevalent [6]. Furthermore, these snails serve as vectors for *S. bovis, S. curassoni,* and *S. mattheei* [7,8], as well as for other trematode species, particularly amphistomes [9,10], which infect livestock. Additionally, some schistosomes and their hybrids, such as *S. mattheei*, transmitted by bulinines, are the causative agents of zoonotic schistosomiasis [8,11,12]. Their predominance and diversification on the African continent also pose interesting biogeographical and evolutionary questions.

Accurate identification of bulinine snails is crucial for understanding schistosomiasis transmission. Historically, identification has relied primarily on morphological characteristics, supplemented by cytogenetic analysis and biochemical assays, such as protein electrophoresis*.* While a few species, such as *B. umbilicatus*, can be identified reliably using conchological features [6], there are no definitive morphological traits that clearly distinguish most *Bulinus* species from one another [13]. The number of bulinine species has fluctuated from more than 120 in early works cited by Wright (1971) [14] to 20 species recognized by Mandahl-Barth (1957) [15], and now to the 37 currently acknowledged, as described by Brown (1994) [6]. These 37 species are categorized into three groups and one complex: the *B. africanus* group (10 species), the *B. forskalii* group (11 species), the *B. reticulatus* group (2 species), and the *B. truncatus/tropicus* complex (14 species) [6]. For convenience, we refer to them hereafter as the *africanus*, *forskalii*, *reticulatus*, and *truncatus/tropicus* species groups in this paper.

Early cytogenetic studies suggested that the first three groups consist solely of diploids (2n = 2 x 18), while the latter includes both diploids and polyploids (4n, 6n, and 8n) [6]*.* Recent molecular studies focusing on species identification, population genetics, and phylogenetics have utilized partial mitochondrial DNA (mtDNA) sequences, such as *cytochrome c oxidase subunit I* (*COX1*) and nuclear ribosomal DNA (rDNA) segments, like internal transcribed spacer (ITS) [16–24]. These studies have provided valuable insights into a broad range of bulinine species across considerable geographic areas, enabled the assembly of near genus-wide phylogenies, and highlighted persistent questions regarding the presence of still-unresolved species groups and inconsistencies between morphological and molecular data. Genomic data are more informative but remain limited [25–28].

In this study, we conducted an integrative analysis of bulinine snails collected from diverse geographical localities across Kenya, including major schistosomiasis-endemic areas. By combining nuclear genome size estimation, complete mitochondrial genome sequencing, and detailed morphometric profiling, we provide novel insights into the cytogenetics, polyploidy, genomics, taxonomy, and evolution of bulinine snails in Africa.

## Materials and methods

### Ethics statement

The collections were approved by the National Commission for Science, Technology, and Innovation (permit numbers P/15/9609/4270 and P/21/9648), the National Environmental Management Authority (permit numbers NEMA/AGR/46/2014 and NEMA/AGR/149/2021), and the Kenya Wildlife Service (permit numbers KWS 0004754 and KWS-0045-03-21).

### Snail collection

The snails for this study were collected in November 2013 and January 2024. Snails in shallow water were collected using long handheld scoops, while a metal dredger was used to collect aquatic plants from deeper waters of Lake Victoria. The snails attached to the aquatic grass were then separated and collected. In each location, the global positioning system (GPS) coordinates were recorded, and photographs of the habitat were taken. Live snails were shipped to the University of New Mexico (UNM) and maintained at the UNM Center for Evolutionary and Theoretical Immunology (CETI). The specimens were assigned temporal codes based on geographical location, but the final species determination for each specimen was based on phylogenetic analyses of mtDNA sequences (see Results section below).

### Flow cytometric analysis of DNA content

DNA measurements were conducted at two different times: first in May 2022 for samples collected in November 2013 and second in April 2024 for samples collected in January 2024. For both measurements, propidium iodide-based flow cytometry was employed. The procedure and experimental design for the two measurements were the same, as described by Bu et al. (2023) [29]. In the first experiment, we used the garden plant hosta (*Hosta plantaginea*) (Praying Hands’ 2-2-2) as a control [29]. In the second experiment, we used the pea (*Pisum sativum*) (seed resource: Gene Bank Dept., CRI Prague-Ruzyne ACCENUMB: 09L010500; Pisum sativum subsp. sativum ACCENAME: Ctirad; ORIGCTY: Czechoslovakia) since the garden plant hosta was unavailable. The DNA content of the pea is 9.02 picograms (pg) (2C DNA content or diploid genome DNA) [30]. To ensure the reliability of the data, the DNA content of the genetically stable homozygous iM line *Biomphalaria glabrata* developed at UNM [31] was measured in both experiments (see details in the Results section).

### DNA extraction, library preparation, and sequencing

The procedures for DNA extraction, quality control, library preparation, and sequencing to generate Illumina 150 bp x 2 paired-end reads and PacBio HiFi CCS reads (high-fidelity circular consensus sequencing) were outlined by Zhang et al. (2024) [32] and Bu et al. (2023) [29], respectively.

### Assembly of mitogenomes

Raw reads were trimmed with Trimmomatic v0.39 to remove low-quality bases and adapter sequences [33]. Four methods were used to assemble the mitogenome from Illumina data: (1) a semi-reference-based assembly with MITOBIM 1.9.1 using an iterative baiting approach [34], (2) *de novo* assembly with NOVOPlasty using a seed-and-extend algorithm [35], (3) reference-guided *de novo* assembly with the specialized toolkit GetOrganelle [36], which employs a baiting and iterative mapping approach, and (4) *de novo* assembly using SPAdes 3.15.1 [37]. If an inconsistency at a particular nucleotide position was found, although very rare, the determination of the nucleotide was based on the majority consensus of the alignments, supported by verifying a functional reading frame. For mitochondrial genome assembly from HiFi reads, we employed the MitoHifi pipeline [38], which is well-suited for long reads with high coverage. HiFi reads were mapped to the reference mitogenome of *B. truncatus* (NC_060795.1) using Minimap2 [39]. The filtered reads were assembled with Hifiasm [40], and the resulting contigs were subjected to BLAST against the reference mitogenome. The final assembly was determined using the four datasets, with additional corrections made based on alignments and annotations.

### Annotation of mitogenomes

Initial mitogenome assemblies were annotated using MITOS2 with RefSeq 63 Metazoa as a reference [41] available from Galaxy Europe [42]. The computational annotation results were used to check the completeness of the mitogenome assemblies. Missing and incomplete gene predictions (including some tRNAs) were corrected manually. Final annotation followed the criteria from Fourdrilis et al. (2018) [43] and Ghiselli et al. (2021) [44], incorporating unique aspects of mitogenome biology, including transcription as polycistronic RNA, the tRNA punctuation model (delimitation of reading frames by tRNA genes or secondary structures), and the completion of stop codons by polyadenylation of mRNA transcripts, as outlined by Zhang et al. (2022) [26]. Mitogenome maps were visualized using SnapGene software (viewer v.7.2.1) (www.snapgene.com).

### Phylogenetic analyses of mitogenome and *COX1* gene sequences

Complete mitochondrial DNA sequences (14,297 bp) and mitochondrial cytochrome c oxidase subunit I (*COX1*) gene sequences (611 bp) were aligned using MUSCLE [45] as performed in Molecular Evolutionary Genetics Analysis (MEGA X) [46]. The *COX1* sequences analyzed corresponded to the standard DNA barcoding region of the gene [47]. Phylogenetic analyses included sequences from multiple *Bulinus* species, with *Biomphalaria glabrata* (iM-line) used as an outgroup. For the *COX1* analysis, taxa included both reported sequences from GenBank and newly generated sequences from this study. The best-fit nucleotide substitution models were selected using the Bayesian Information Criterion (BIC) implemented in MEGA X: T92 + G + I for *COX1* [48] and GTR + G + I for complete mtDNA [49]. Maximum Likelihood (ML) phylogenetic trees were constructed in MEGA X using the identified best-fit models, with branch support assessed using 1,000 bootstrap replicates [50]. Trees are presented as phylograms with branch lengths proportional to evolutionary distance.

### Morphometric analysis

**Fig 1:**
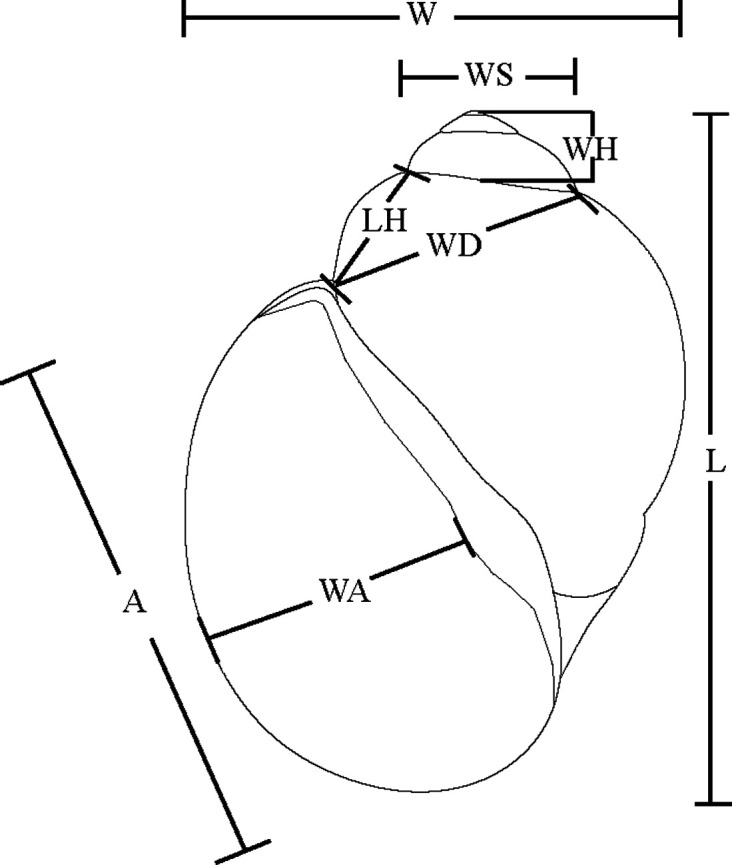
Schematic representation of snail shell morphometrics. The abbreviations are provided in the Materials and Methods section.

Shell morphometric analysis was conducted using a landmark-based approach by orienting each shell with the aperture facing the observer (Fig 1). Eleven linear shell measurements were recorded using a digital caliper (0.01 mm precision). The measurements included shell length (L), shell width (W), aperture height (A), aperture width (WA), whorl spacing (WS), diagonal shell width (WD), body whorl height above the aperture (LH), and whorl height (WH). Raw measurements were compiled into structured Excel spreadsheets, and seven morphometric ratios were derived to quantify shell proportions: shell elongation (L/W), aperture proportions (A/L), spire development ((L-A)/L), body whorl expansion (LH/L), relative aperture width (WA/WS), diagonal whorl expansion (WD/W), and sutural spacing (WH/L).

Data preprocessing, taxonomic grouping, and descriptive statistics (mean ± SD) were performed in RStudio (version 2023.12.0 + 369) using the tidyverse package [51]. To investigate multivariate morphological patterns, Principal Component Analysis (PCA) was implemented via FactoMineR [52], with variance contributions from principal components reported. Pairwise relationships between shell variables were visualized using bivariate plots generated with ggplot2 [53] and RColorBrewer [54].

## Results

### Snail sampling in Kenya

**Fig 2:**
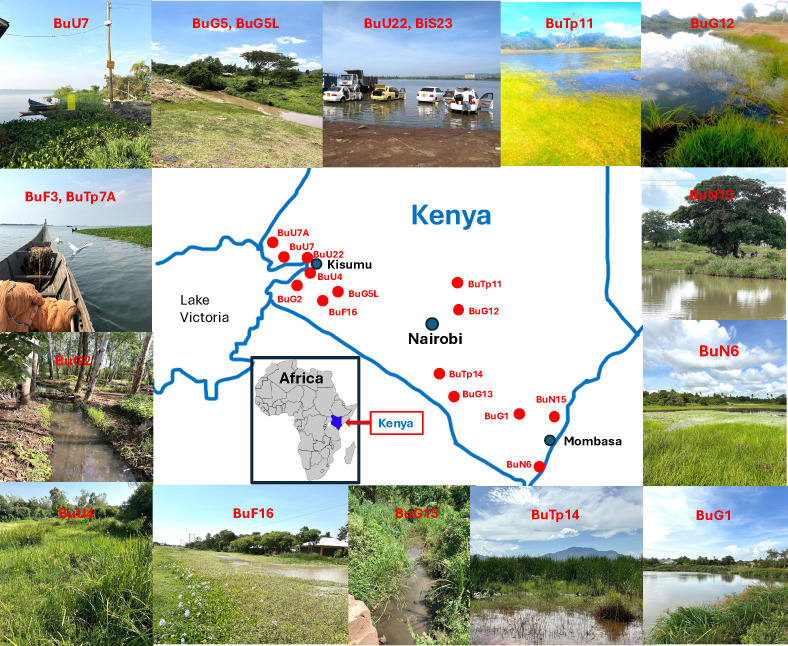
Geographical localities and natural habitats of snails collected in Kenya. The approximate localities are indicated. Detailed information for each locality (GPS) and its corresponding code is provided in Table 1. The photos in this figure were taken by the authors.

In two samplings conducted 10 years apart (November 2013 and January 2024), 14 localities were sampled, as shown in Fig 2. For convenience, each locality was assigned a code corresponding to its operational taxonomic group, as detailed in Fig 2 and Table 1. Please note that not all specimens were available for all three analyses (nuclear genome size, mitogenome, and morphometrics) due to limitations in specimen availability.

**Table 1:** DNA content (C-value) and relevant information of the analyzed samples in this study.

Species	Code	Locality	GPS	C-value (pg)	Average	Date of sampling and analysis
*B. ugandae*	BuU4	Tiengre	00^0^5’27”S 34^0^ 42’14”E	1.44	1.42	January 2024 April 2024
				1.42		
				1.41		
*B. ugandae*	BuU7	Dunga beach	00^0^8’41”S 34^0^ 44’10”E	1.46	1.46	January 2024 April 2024
				1.54		
				1.39		
*B. ugandae*	BuU22	Car Wash site, Kisumu	00^0^05’45.00”S 34^0^ 44’57.69”E	1.29	1.33	November 2013 May 2022
				1.30		
				1.39		
*B. nasutus*	BuN6	Mwamagogo Dam	4^0^27’58”S 39^0^ 27’58”E	1.52	1.41	January 2024 April 2024
				1.34		
				1.38		
*B. nasutus*	BuN15	Mazeras quary pond	3^0^55’52”S 39^0^33’15’E			January 2024. Mitogenome analysis
*B. globosus*	BuG1	Kinango dam	4^0^8’12”S 39^0^ 18’60’E	1.42	1.43	January 2024 April 2024
				1.55		
				1.32		
*B. globosus*	BuG2	Migosi ditch	00^0^4’19”S 34^0^ 46’32”E	1.92	1.98	January 2024 April 2024
				2.04		
				1.88		
*B. globosus*	BuG5	Asao stream	00^0^19’5.50”S 35^0^ 0’24.99”E	1.87	1.88	November 2013 May 2022
				1.89		
				1.88		
*B. globosus*	BuG5L	Asao stream	00^0^19’5.50”S 35^0^ 0’24.99”E	1.73	1.86	January 2024 April 2024
				2.00		
				1.88		
*B. globosus*	BuG12	Kwa Katiwa dam	1^0^18’59”S 37^0^18’00”E	1.71	1.50	January 2024 April 2024
				1.44		
				1.56		
*B. globosus*	BuG13	Eldoro Taveta	3°28’53.4“S 37°41’36.4”E	1.34	1.36	January 2024 April 2024
				1.37		
				11.34		
*B. forskalii*	BuF3	Usenge beach	00^0^4’22”S 34^0^ 3’33”E	0.76	0.76	February 2024 April 2024
				0.75		
				.076		
*B. forskalii*	BuF16	Rabuor-Ahero	00^0^9’19”S 34^0^ 51’2”E			January 2024. Mitogenome analysis
*B. tropicus*	BuTp7A	Usenge beach	00^0^4’22”S 34^0^ 3’33”E	1.34	1.32	January 2024 April 2024
				1.32		
				1.30		
*B. tropicus*	BuTp11	Kisukioni dam	1013’27”S 37016’20”E			January 2024. Mitogenome analysis
*B. tropicus*	BuTp14	Lake Jipe	3°33’60.0“S 37°46’01.2”E	1.00	1.03	January 2024 April 2024
				1.04		
				1.06		
*B. truncatus*	BuT19	Egypt		1.87	1.82	May 2022
				1.89		
				1.71		
*Bi. sudanica*	BiS23	Car wash site Kisumu	00^0^05’45.00”S 34^0^ 44’57.69”E	1.01	1.00	November 2013 May 2022
				1.00		
				1.00		
*Bi. glabrata* (iM line)	BiG24	UNM		1.10	1.11	April 2024
				1.11		
				1.11		

Note: Three DNA content samples and associated data, including iM line *Bi. glabrata* (1.09 pg), BB02 *Bi. glabrata* (1.02 pg) and *Bi. pfeifferi* (0.91 pg) published by Bu et al., 2023 [29] are coded as BiG25, BiG26, and BiP27, respectively. Some specimens were archived in the UNM Museum of Southwestern Biology (MSB) with MSB host voucher numbers as follows: BuGN6 (25687), BuG2 (25681), BuG5L (25682), BuF3 (25680), BuTp7A (25683), BuTp9 (25685), BuTp10 (25684), and BuTp14 (25686).

### Flow cytometric analysis of nuclear DNA contents

**Fig 3:**
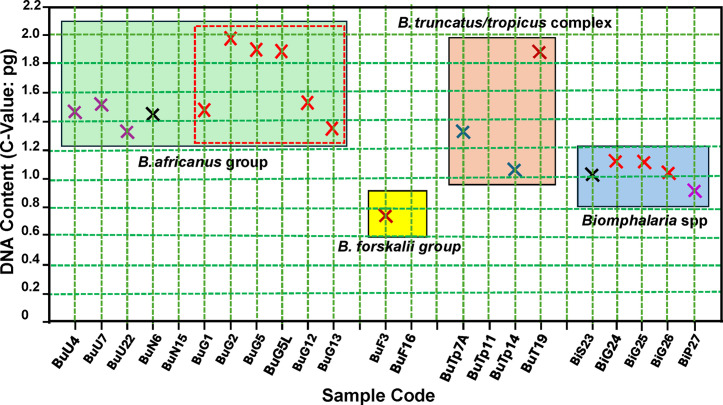
Genome sizes (C-value) of *Bulinus* and *Biomphalaria* snails. Detailed information for the codes is provided in Table 1. Codes Bi25, Bi26, and Bi27 represent the iM line *Bi. glabrata* (10.9 pg), BB02 *Bi. glabrata* (1.02 pg), and *Bi. pfeifferi* (0.91 pg), respectively, as reported by Bu et al. (2023) [29]. All samples of *B. globosus* are enclosed by dotted lines.

For each code, three snails were individually measured using flow cytometry. A total of 16 coded samples were analyzed, including 14 from the genus *Bulinus*, representing three bulinine groups: *B. africanus*, *B. forskalii*, and *B. truncatus/tropicus*, along with 2 from the genus *Biomphalaria* (Table 1). Three specimens of *Biomphalaria* reported by Bu et al. (2023) [29] were included to add perspective to the analysis. All DNA contents presented in this study pertain to the C-value, which refers to the amount of DNA in a haploid cell. Table 1 presents the C-values of individual snails and their averages. Fig 3 displays the average C-values of snails from the *africanus*, *forskalii*, and *truncatus/tropicus* groups, as well as *Biomphalaria* spp. The smallest genome was 0.76 pg from *B. forskalii* (BuF3), while the highest DNA content, 1.98 pg, was observed in a specimen of *B. globosus* (BuG2), with similar amounts noted in *B. truncatus* (BuT19: 1.82 pg) and two other *B. globosus* specimens (BuG5L: 1.86 pg; BuG5: 1.88 pg). The DNA contents of a UNM-maintained homozygous iM line of *Bi. glabrata* [31] from this study (code: BiG24) (Table 1) and the same line previously measured and reported (BiG25) [29] were consistent (1.11 pg vs. 1.09 pg: p = 0.58). Moreover, two field-collected *B. globosus* samples (BuG5L and BuG5) from the same geographical locality (Asao, Kisumu), taken 10 years apart and measured at different times, yielded consistent results (1.88 pg vs. 1.86 pg: p = 0.80) (Table 1). These findings support the reliability of the data presented in this study.

### Determination of complete mitogenome sequences and structure

Complete mitochondrial genomes (mitogenomes) from 14 bulinine specimens were sequenced, assembled, and annotated (S1 Table). The list of specimen codes and their GenBank accession numbers (in parentheses) is as follows: BuU4 (PV483366), BuU7 (PV483367), BuN6 (PV483368), BuN15 (PV483369), BuG1 (PV483370), BuG2 (PV483371), BuG5L (PV483372), BuG12 (PV483373), BuG13 (PV483374), BuF3 (PV483375), BuF16 (PV483376), BuTp7A (PV4833677), BuTp11 (PV483378), and BuTp14 (PV483379). The species and locality corresponding to the specimen codes are listed in Table 1.

Each mitogenome consists of 37 genes, including 13 protein-coding genes (PCGs), 22 transfer RNA (tRNA) genes, and 2 ribosomal RNA (rRNA) genes. The arrangement and order of the PCGs are consistent across all mitogenome sequences determined in bulinines, including those published previously [26]. A difference was observed in the location of tRNA-D (tRNA-Asp), dividing the 14 mitogenomes into two types: DCF (Asp-Cys-Phe) and CF (Cys-Phe). The distinction between the two types lies in the location of D; in the CF type, D is positioned downstream of the mitogenome between Y and W (YDWGHQL), whereas in the DCF type, DCF are grouped together. All species of the *africanus* group belong to the CF type, while species of the *forskalii* and *truncatus/tropicus* groups belong to the DCF type (S1 Data).

### Phylogenetic analyses of mitogenome sequences

**Fig 4:**
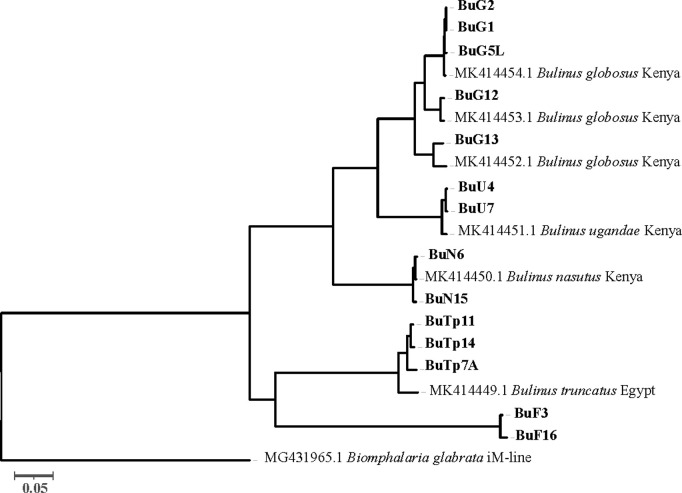
Maximum Likelihood phylogram of complete mitochondrial genome sequences in *Bulinus* snails. The tree is presented as a phylogram with branch lengths proportional to evolutionary distance (scale bar = 0.05 substitutions per site). Phylogenetic relationships were inferred using the GTR + G + I substitution model with 1,000 bootstrap replicates implemented in MEGA X [46]. A total of 21 complete mitogenome sequences (14,297 bp) were analyzed: 14 from the current study (BuF3, BuF16, BuTp7A, BuTp11, BuTp14, BuN6, BuN15, BuU4, BuU7, BuG1, BuG2, BuG5L, BuG12, and BuG13) and 7 published sequences, including 6 from Zhang et al. [26] and *Biomphalaria glabrata* (iM-line; MG431965.1) used as an outgroup. The tree demonstrates the phylogenetic relationships among major *Bulinus* species groups: the *africanus* group (including *B. globosus*, *B. ugandae*, and *B. nasutus*), the *truncatus/tropicus* complex, the *forskalii* group, and the *reticulatus* group. Branch lengths reflect the degree of evolutionary divergence between taxa.

**Fig 5:**
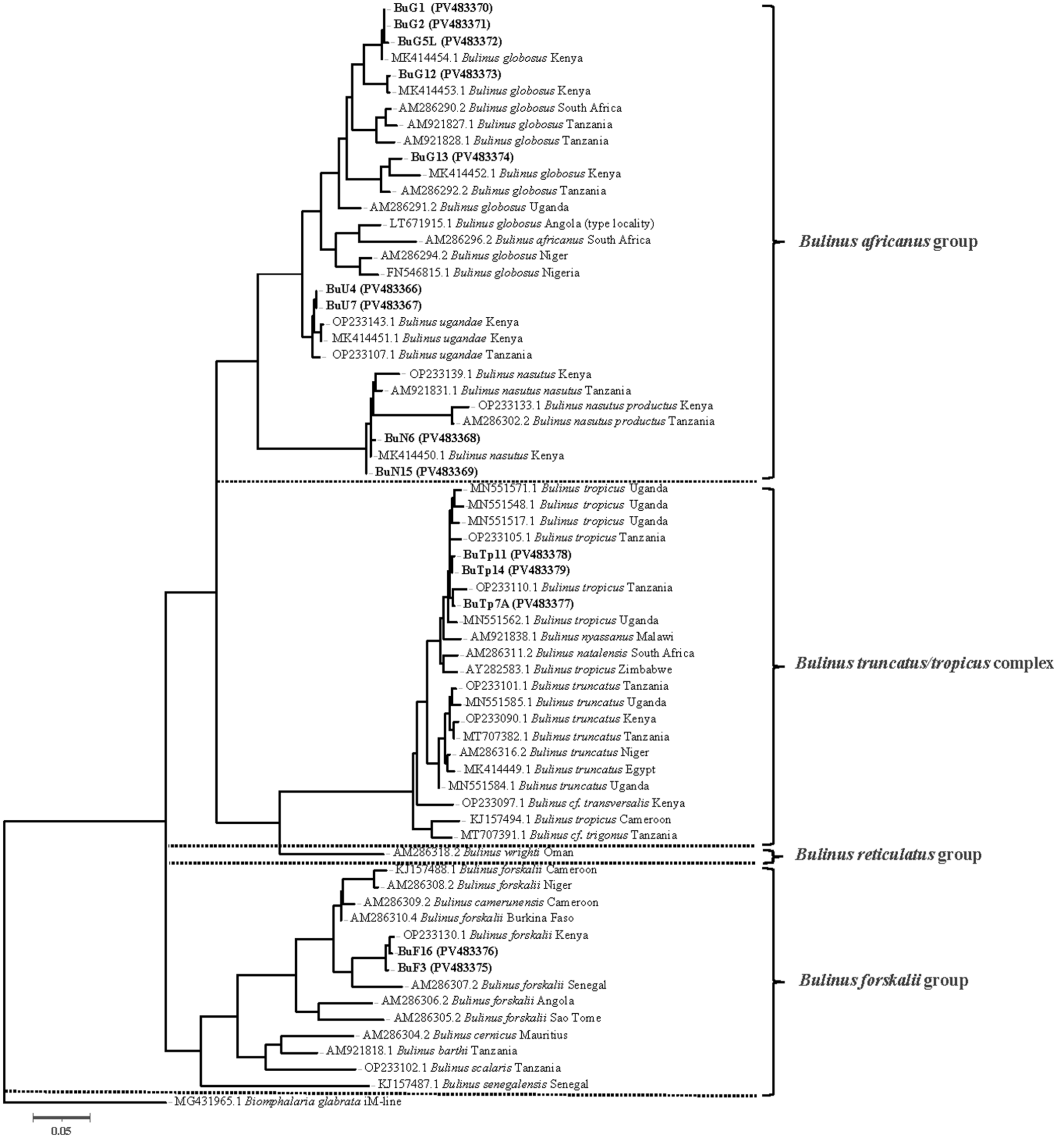
Maximum Likelihood phylogram of the mitochondrial *COX1* gene in *Bulinus* snails from Africa. The tree is presented as a phylogram with branch lengths proportional to evolutionary distance (scale bar = 0.05 substitutions per site). Phylogenetic relationships were inferred using the T92 + G + I substitution model with 1,000 bootstrap replicates implemented in MEGA X [46]. The analysis included 67 *COX1* sequences (611 bp): 14 newly generated sequences from this study (indicated by specimen codes with GenBank accession numbers in parentheses) and 53 published sequences from GenBank representing several African *Bulinus* species and geographic localities. *B. glabrata* (iM-line; MG431965.1) was used as an outgroup.

A total of 20 complete bulinine mitogenome sequences, including six from a previous report [26], were phylogenetically analyzed (Fig 4). The *africanus* and *truncatus/tropicus* species groups appear to cluster together. Two *B. forskalii* mitogenomes represent the first complete mitogenome sequences from the *forskalii* species group and cluster with the other two groups. Phylogenetic analysis of mitochondrial *COX1* gene sequences supports the topology of the complete mtDNA tree (Fig 5). In the *africanus* group, *B. globosus* is more closely related to *B. ugandae* than to *B. nasutus* in both mitogenome and COX1 analyses. The phylogenetic analyses indicate that specimen BuTp7A, collected from deep water in Lake Victoria, is *B. tropicus* (see Discussion below). The divergence between *B. truncatus* and *B. tropicus* appears significant, although their morphology and size are very similar.

### Morphometric analysis

**Fig 6:**
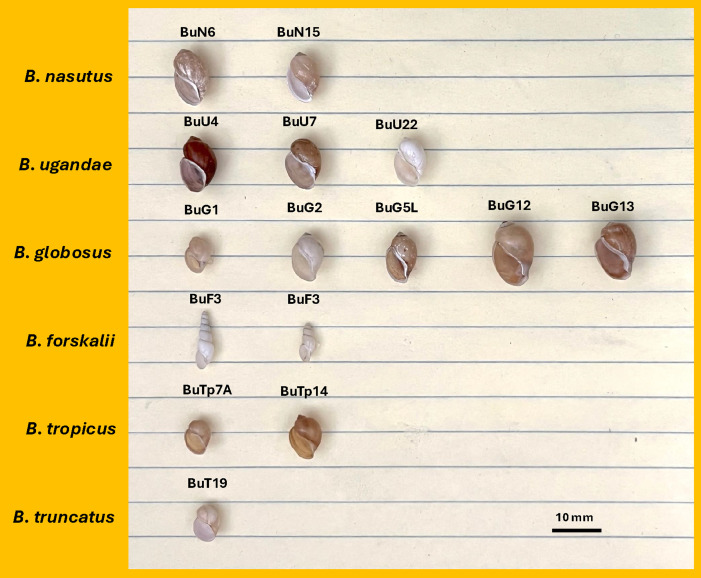
Shell morphology of bulinine specimens. Details about the specimen codes, species, and their localities are provided in Table 1. The photograph was taken by S-MZ.

**Fig 7:**
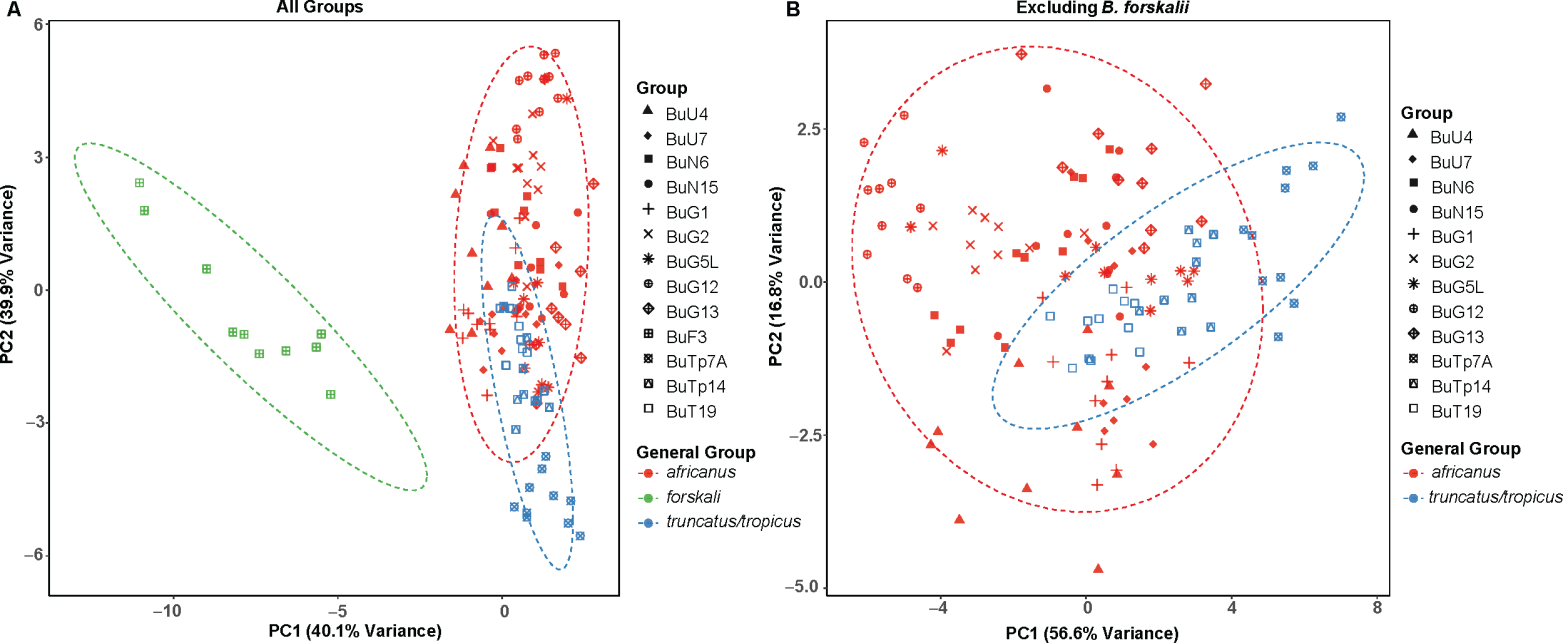
Principal component analysis (PCA) of shell morphometric variables for *Bulinus* species. (A) PCA plot including all *Bulinus* groups, showing the first two principal components that explain 40.8% and 38.7% of the total variance, respectively. (B) PCA plot excluding *B. forskalii* (BuF3), with the first two principal components explaining 57.3% and 15.4% of the total variance, respectively. Shapes represent different *Bulinus* groups, with symbol colors corresponding to taxonomic groups (*africanus*, *forskalii*, and *truncatus/tropicus*).

Morphometric analysis revealed substantial challenges in distinguishing among *Bulinus* species. As shown in Fig 6, visual examination of shells did not reliably differentiate the species, especially within a given species group. Further analyses of various shell morphometrics (S2 and S3 Tables) indicated that, despite statistically significant ANOVA differences across all groups (all *p* < 0.01; S4 Table), only the *forskalii* group (e.g., *B. forskalii*) was clearly separated from the *africanus* and *truncatus/tropicus* groups in PCA analysis (PC1: 40.1% variance; Fig 7A). The latter two groups exhibited significant overlap (Fig 7B), even after excluding *B. forskalii* (PC1: 56.6%, PC2: 16.8%). Within the *truncatus/tropicus* group, tetraploid *B. truncatus* and diploid *B. tropicus* could not be distinguished morphologically (Fig 7B and S3 Table). Similarly, within the *africanus* group, species such as *B. globosus*, *B. ugandae*, and *B. nasutus* overlapped in shell dimensions (L, W, A) and aperture characteristics (WA, WS) (S1 and S2 Figs). Additionally, *B. globosus* specimens from different localities showed no clear morphological differentiation (S3 Table), with overlapping distributions in bivariate plots (S1 and S2 Figs).

## Discussion

### Unexpected variation in nuclear genome sizes

In the family Planorbidae, which includes *Bulinus* and *Biomphalaria* [6], the basic chromosome number is n = 18 [55]. *Biomphalaria* snails are diploid, as confirmed by cytogenetic studies, DNA content analysis, and whole genome sequencing [29,31,56–59]. Some authorities consider *Bulinus* snails to be in a separate family, the Buliniade, along with *Indoplanorbis* and a few other genera [60]. *Indoplanorbis* is also known to contain a haploid number of 18 chromosomes [61]. In *Bulinus*, chromosome numbers and instances of polyploidy were determined through chromosome counting conducted in the 1960s and 1970s [e.g., 62–65]. These studies provided invaluable information for understanding the cytogenetics of *Bulinus* snails but were not without limitations [64]. Due to technical constraints at that time and the challenges of collecting, transporting, and maintaining live snails, specimens collected in the field were preserved in chemical fixatives (e.g., Carnoy’s solution) and transported to the laboratory for cytogenetic analyses. These chromosome numbers were established from meiotic figures at the diakinesis stage of gonadal tissue (ovotestes). Karyotype analysis based on somatic metaphase chromosomes, which requires tissues or organs from live snails, was performed in the 1980s on only three bulinine species, *B. truncatus*, *B. tropicus*, and *B. natalensis* [56,66,67].

Given the challenge of obtaining somatic metaphase chromosome numbers or conducting karyotype studies, DNA content analysis is a viable option, particularly for polyploid species. Generally, within a closely related group of species, there is a correlation between chromosome number and genome size or DNA content [68,69]. Relative DNA contents among closely related species provide useful indications of ploidy levels and changes in genome sizes. The first effort to measure nuclear DNA content, focusing on three *Bulinus* species groups: *africanus*, *forskalii*, and *truncatus/tropicus* was performed using flow cytometry and presented in this study. The smallest genome size was found in *B. forskalii*, where all species in the *forskalii* group are diploid. Among the representatives of the *truncatus/tropicus* group we examined, in concordance with cytological studies [62–64], two general types of genome sizes were observed, with interspecific and intraspecific variation: tetraploid *B. truncatus* had significantly more DNA content than diploid *B. tropicus*. *B. truncatus*, including a strain from the type locality in Egypt maintained at the Biomedical Research Institute (BRI: www.afbr-bri.org) for decades, has been confirmed to have a total of 72 chromosomes by multiple cytogenetic studies [56,65,70–72], serving as a reliable reference for genetic and genomic studies in *Bulinus* snails.

An unexpected finding was noted in the *B. africanus* group, particularly for the taxon *B. globosus*, which is increasingly recognized as a likely species complex [22,24,26]. All species assayed in this group, including *B. globosus*, *B. ugandae*, and *B. nasutus*, are believed to be diploid [6]. However, the variation in DNA content (1.33 to 1.98 pg) is notably high, similar to that of the *truncatus/tropicus* group (1.03-1.82 pg), which contains both diploid (*B. tropicus*) and tetraploid (*B. truncatus*) species. The DNA content of *B. globosus* ranges from 1.43 to 1.98 pg, with the highest value (1.98 pg) slightly exceeding that of tetraploid *B. truncatus* (1.82 pg). The C-values of two *B. globosus* samples from western Kenya (BuG2: 1.98 pg; BuG5L: 1.86 pg) are higher than the C-value of *B. globosus* from the eastern coastal Kinango dam (BuG1: 1.43 pg) and the central regions, Kwa Katiwa dam (BuG12: 1.50 pg) and Eldoro in Taveta (BuG13: 1.36 pg). The high variability in genome size within the *africanus* group, particularly among different specimens of *B. globosus*, suggests dynamic genomic variation and adds further support to the hypothesis that *B. globosus* may comprise multiple or cryptic species [22,26].

*B. ugandae* (1.33-1.46 pg) and *B. nasutus* (1.41 pg) have a small range variation in genome size, but their genome sizes are larger than those of diploid species such as *Biomphalaria* spp. (0.91-1.11 pg) or *B. forskalii* (0.76 pg) and *B. tropicus* (1.03-1.32 pg), while being smaller than that of the tetraploid *B. truncatus* (1.82 pg). Notably, with the exception of *Bulinus forskalii*, the genome sizes of known diploid *Bulinus* snails are larger and more variable than those of the measured *Biomphalaria* species (Fig 2), despite both sharing the same basal chromosome number of n = 18, characteristic of the family Planorbidae [55]. Early-diverging South American representatives of *Biomphalaria* [73] are as yet unknown with respect to genome size and variability. With the evidence currently in hand, some representatives of *Bulinus* have subsequently undergone processes (genome expansion and/or whole genome duplication) that have resulted in much greater variability in genome size, including puzzling variations among species generally considered diploid.

### Genome expansion and whole genome duplication (polyploidization)

The profound genome size variation uncovered in the current study suggests that bulinine snails, particularly the *africanus* group, have undergone genome expansion, whole genome duplication (polyploidization), or both processes.

Genome expansion or variation in genome size without changes in ploidy levels has been documented in many eukaryotes [74], including shrimp of the genus *Synalpheus* [75,76], flour beetles of the genus *Tribolium* [77], the fruit fly *Drosophila* [78], and the rotifer *Brachionus plicatilis* [79]. The genome size of *B. globosus* is comparable to that of the tetraploid *B. truncatus*, and significantly higher than that of recognized diploid species such as *B. forskalii*, *B. tropicus*, and *Biomphalaria* spp. If the *B. africanus* group species are indeed true diploids, they must have undergone significant genome expansion (i.e., increased chromosome sizes without changing chromosome numbers). Furthermore, given the known genome sizes of three species of *Biomphalaria* (*Bi. glabrata, Bi. pfeifferi, and Bi. sudanica*) and *B. forskalii*, it appears that most diploid bulinines have undergone some degree of genome expansion, while *B. forskalii* may have experienced slight genome depletion.

Polyploidization resulting from whole genome duplication has been found in animals, including the New Zealand snail *Potamopyrgus antipodarum* [80–82]. In bulinine snails, it has been documented only in the *truncatus/tropicus* group, where tetraploidy, hexaploidy, and octoploidy have been recorded [6]. Our genome size data imply that the *africanus* group may possess triploid and tetraploid forms, assuming that homologous chromosome sizes are conserved across the family Planorbidae. Triploidy has not been reported for any *Bulinus* species, while tetraploidy has only been found in the *truncatus/tropicus* group. Although early cytogenetic studies indicate that there are no polyploids in the *africanus* group [6], a recent population genetics study suggests that polyploidy may be present in *B. globosus*, a taxon from the *africanus* group, in Kenyan populations [24]. To clarify these intriguing questions, further investigations are needed to analyze nuclear DNA content and/or somatic chromosomes in various populations, particularly within the *B. globosus* species complex.

Polyploidy can influence an organism’s phenotypes, including its refractoriness to parasites. Increased allelic diversity may enable hosts to recognize a broader range of parasites [83–85]. Alternatively, the combination of subgenomes from different species may alter gene expression, also affecting the degree of parasite resistance [86–89]. Triploid *P. antipodarum* have greater resistance to allopatric parasites than diploids, suggesting the advantages of increased ploidy for hosts facing coevolving parasites [83]. In bulinines, the host-trematode (including schistosomes) systems are highly complex; several trematode species, including multiple schistosome parasites, as described in the Introduction section, employ bulinines as hosts. For the human parasite *S. haematobium*, some bulinine species or ecotypes are susceptible, while others are not [6,90]. The role of genome duplication and expansion in influencing infection outcomes presents a compelling question in evolutionary biology and schistosomiasis transmission.

The origin of polyploidy in *Bulinus* remains unknown. Diploid and tetraploid species from the *B. truncatus/tropicus* complex are widely distributed across Africa, while hexaploid and octoploid forms are confined to the highlands of Ethiopia [65]. In Kenya, diploids and tetraploids, but no hexaploids or octoploids, have been found in the highlands [1,70]. The tetraploid *B. permembranacea* found in the Kenyan highlands is morphologically different from *B. truncatus* found in other regions, suggesting at least two independent origins of tetraploidy in Africa [70]. Our findings add further complexity to the understanding of bulinine snails in Africa. Using a comparative karyotype approach, Goldman et al. (1983) hypothesized a hybrid origin for tetraploid *B. truncatus* [67]. Modern genomic technologies, such as subgenome analysis that have been applied in other polyploid organisms [91–94], may yield valuable insights into the origin of polyploidy in bulinines.

### Conservation and variation of complete mitogenomes

Our study adds 14 new complete and annotated bulinine mitogenome sequences to public databases, bringing the total to 20 complete and annotated bulinine mitogenome sequences available to date [26]. We demonstrate that the protein-coding genes (PCGs) of all bulinine mitogenomes are well conserved. However, a difference was noted in the location of tRNA-D, which divides the *Bulinus* snails we sampled into two types: DCF and CF, as noted in our previous study [26]. In terms of gene organization, the sampled members of the *forskalii* and *truncatus/tropicus* groups share the same structure, referred to as the DCF type, while the *africanus* group exhibits the CF type. This difference results from the open reading frame (ORF) of the *ND4* gene terminating with an incomplete stop codon (T--) followed by an inverted repeat (TAACAGAATTCTGTTA), forming a hairpin structure at the 3’ end of the transcript in the DCF group. The *forskalii* and *truncatus/tropicus* groups are more closely related to each other than to the *africanus* group from a mitogenome structure perspective.

### Implications for phylogenetic analyses

**Fig 8:**
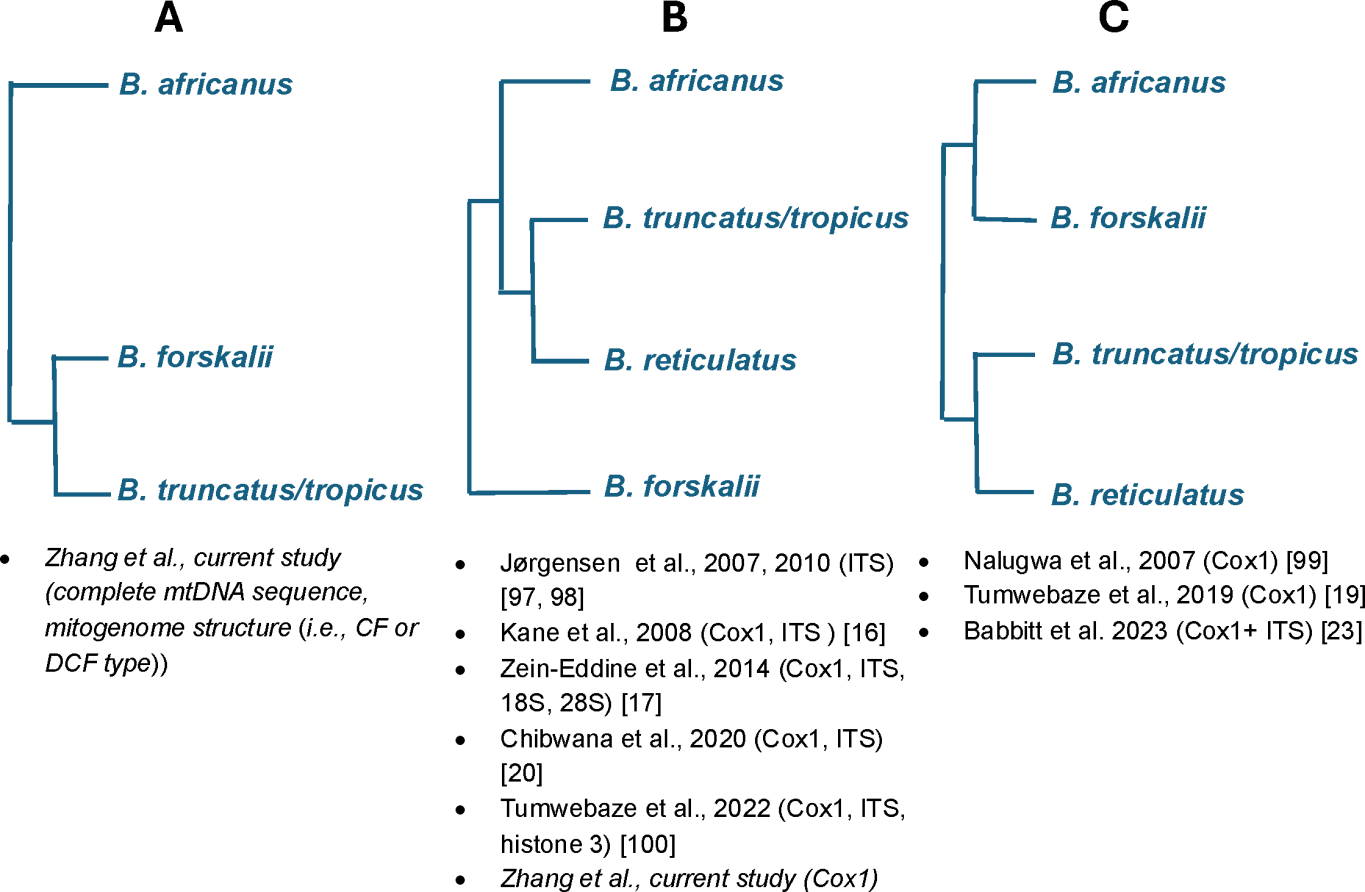
A summary of the relationship patterns among bulinine species groups based on the current study and published papers. References for each type of relationship (A, B, and C) are provided. The species studied from the *B. reticulatus* group is normally *B. wright*, as there are only two species (*B. reticulatus* and *B. wright*) in this group.

The availability of twenty *Bulinus* and six *Biomphalaria* mitogenomes [26, 95, this study] provides valuable genetic markers for population genetics and phylogenetic analyses. This enables a more comprehensive investigation of mitogenome evolution in schistosomiasis-transmitting planorbid snails, which are responsible for most global schistosomiasis transmission. Our full-length mtDNA tree indicated that the *B. forskalii* group is more closely related to the *B. truncatus/tropicus* complex than to the *B. africanus* group (Figs 4 and 8A), aligning with the pattern revealed by mitogenome structure (see above). This relationship, despite the exclusion of the *B. reticulatus* group, differs from patterns revealed by *COX1* and ITS (Fig 8B and 8C) [16,17,19,20,23,96–100]. Our *COX1* tree also indicated that species from the *B. africanus* group cluster with species from the *B. truncatus*/ *tropicus* group (Figs 5 and 8B) [16,17,20,97,98,100], which is different from the finding that the *B*. *forskalii* and *B. truncatus/tropicus* are more closely related to each other than to the *B. africanus* group (Fig 8C) [19,23,99]. These variable patterns among the species groups within *Bulinus* suggest that establishing species concepts in *Bulinus* requires more evidence. Employing whole genome sequencing, rather than focusing on a few loci, may provide a more robust pattern of relationships.

The phylogenetic analyses also support the complexity of the taxon *B. globosus* as revealed by nuclear genome size variation in this study and previous mtDNA analyses [22,24,26]. It also helps clarify ambiguous species. The specimen BuTp7A, found in the deep waters of Lake Victoria, is much smaller than *B. tropicus* but similar in size to *B. truncatus*. Morphometric analysis cannot easily differentiate the two species. In Lake Victoria, three species with type localities in the lake are present: *B. ugandae* (2n = 36), *B. transversalis* (2n = 72), and *B. trigonus* (2n: unknown) [6]; however, our phylogenetic analysis indicates that BuTp7A is closer to *B. tropicus* than to these three species. This is surprising, as *B. tropicus* has been commonly found elsewhere but not in deep waters. Furthermore, the DNA content of BuTp7A (1.32 pg) is higher than that of *B*. *tropicus* (BuTp14; 1.03 pg), collected from Lake Jipe, but smaller than that of tetraploid *B. truncatus* (1.82 pg). These findings suggest a complex relationship within the *B. truncatus/tropicus* group, which includes diploid, tetraploid, hexaploid, octoploid, and possibly other polyploids such as triploids.

### Limitations of morphometric analysis

Among the four species groups, the *forskalii* group is easily identified due to its distinct long shell shape. Differentiating between *B. forskalii* and *B. senegalensis*, both from the *forskalii* group using morphometric analysis, is feasible, mainly due to the different whorl shapes in these species [101]. The challenge lies with the remaining *africanus* and *truncatus/tropicus* groups, which consist of 24 species and play a crucial role in schistosomiasis transmission. Similar difficulties in differentiating *B. africanus* group species using shell morphology alone have been reported [102]. Nevertheless, caution is warranted in morphological analysis, as misidentifications from morphological data can lead to misinformation in molecular data in public databases. It is critical to integrate molecular data for reliable species identification, particularly within taxonomically challenging groups where shell morphology exhibits high variability or overlap among species.

### Bulinine species and species identification

In bulinine snails, high morphological variation, the lack of clear conchological characteristics, and the existence of ecotypes and intermediate forms between species make accurate species identification highly challenging. Early cytogenetic work relied on meiotic figures of the ovotestis and requires verification through DNA content and somatic chromosome analysis. While these snails are polyploid, it remains uncertain whether polyploidy can serve as a criterion for species distinction, given that *B. permembranaceus* (4n), *B. hexaploidus* (6n), and *B. octoploidus* (8n)—the polyploid complex found in Ethiopia—were primarily named based on their polyploid characteristics [6,65]. It is common for a single species to exhibit multiple ploidy levels [103]. For example, the New Zealand mud snail *P. antipodarum* has diploid, triploid, and tetraploid forms; yet, they all belong to one species [80–82]. The freshwater fish *Misgurnus anguillicaudatus* has diploid, triploid, tetraploid, pentaploid, and hexaploid forms in natural populations [104]. It seems that ploidy level may not be a reliable characteristic for species identification. At the molecular level, protein electrophoresis was previously employed to assist in species identification. For example, the primary evidence for the distinct species *B. browni* and *B. hightoni* from Kenya was established by protein electrophoretic patterns [6,105,106]. However, caution should be exercised regarding protein expression patterns, as they are controlled by gene regulation, which is highly influenced by ecosystems, environments, and developmental stages. For molecular markers such as proteins and mtDNAs, which have been commonly used, it is unclear what level of genetic differentiation can be considered species level. Furthermore, it is uncertain which molecular marker—mtDNA or nuclear marker—is best to use, given the discordance found between nuclear and mitochondrial genes (mitonuclear discordance) in many species [107–110]. Although these issues are being addressed, defining species remains a challenge, especially since they exhibit hermaphroditism. A combined study of datasets from the nuclear genome, mitochondrial genome, and morphology, as shown in this study, revealed unexpected findings and raised unanswered questions, further highlighting the complexity of bulinine snails in Africa. More comprehensive investigations from various biological perspectives, utilizing modern technologies such as genomics and AI-assisted 3D analysis, while considering specimens from type localities, are necessary to address these complex questions.

## Conclusion

We measured nuclear DNA content, determined complete mitogenomes, and analyzed morphometric data of bulinine snails from three *Bulinus* species groups collected across various localities in Kenya. We uncovered significant variation in DNA content among bulinine specimens, suggesting genome expansion, whole genome duplication (polyploidization), or both in the *B. africanus* group—phenomena that were previously unrecognized, with the mechanisms still unknown. Adding 14 new complete mitogenome sequences enhances our understanding of comparative mitogenomics and provides valuable resources for studies in taxonomy, evolution, population genetics, phylogenetics, and epidemiology. Both genome size and mitogenome analyses indicate that *B. globosus* may comprise multiple or cryptic species (i.e., the *B. globosus* species complex). Our findings confirmed that morphometric data alone are inadequate for reliably identifying *Bulinus* species, reiterating the need for molecular approaches in bulinine identification. This study also raises significant questions about defining and identifying bulinine species as well as the relationships among species groups within the genus *Bulinus*. Analyzing all three types of data simultaneously highlights the complexity of bulinine snails, not only in Kenya but also across the African continent. Further studies are needed to focus on these important yet understudied species, which may enhance our understanding of bulinine biology and disease transmission.

## Supporting information

S1 Table Annotation of 14 complete mitogenomes. (XLSX)

S2 Table Raw shell morphometric measurements (mm) and derived ratios. (XLSX)

S3 Table Mean ± standard deviation (SD) of shell morphometric parameters and ratios. (XLSX)

S4 Table ANOVA results for shell morphometric variables. (XLSX)

S1 Fig Bivariate relationships of shell morphometric variables among *Bulinus* species. (TIFF)

S2 Fig Bivariate relationships of shell morphometric variables among *Bulinus* species after excluding *B. forskalii.* (TIFF)

S1 Data Map and SnapGene file of 14 bulinine mitogenomes. (ZIP)

## References

[1] Brown DS, Shaw KM. Freshwater Snails Of The *Bulinus* Truncatus/tropicus Complex In Kenya: Tetraploid Species. J Mollus Stud. 1989;55(4):509–32. doi: 10.1093/mollus/55.4.509

[2] van der Werf MJ, de Vlas SJ, Brooker S, Looman CWN, Nagelkerke NJD, Habbema JDF, et al. Quantification of clinical morbidity associated with schistosome infection in sub-Saharan Africa. Acta Trop. 2003;86(2–3):125–39. doi: 10.1016/s0001-706x(03)00029-9 12745133

[3] Hotez PJ, Kamath A. Neglected tropical diseases in sub-saharan Africa: review of their prevalence, distribution, and disease burden. PLoS Negl Trop Dis. 2009;3(8):e412. doi: 10.1371/journal.pntd.0000412 19707588 PMC2727001

[4] Adenowo AF, Oyinloye BE, Ogunyinka BI, Kappo AP. Impact of human schistosomiasis in sub-Saharan Africa. Braz J Infect Dis. 2015;19(2):196–205. doi: 10.1016/j.bjid.2014.11.004 25636189 PMC9425372

[5] Phillips AE, Gazzinelli-Guimarães PH, Aurelio HO, Dhanani N, Ferro J, Nala R, et al. Urogenital schistosomiasis in Cabo Delgado, northern Mozambique: baseline findings from the SCORE study. Parasit Vectors. 2018;11(1):30. doi: 10.1186/s13071-017-2592-8 29316983 PMC5761122

[6] Brown DS. Freshwater snails of Africa and their medical importance. Second ed. London: Taylor & Francis Ltd. 1994.

[7] Aula OP, McManus DP, Jones MK, Gordon CA. Schistosomiasis with a Focus on Africa. Trop Med Infect Dis. 2021;6(3):109. doi: 10.3390/tropicalmed6030109 34206495 PMC8293433

[8] Stothard JR, Juhász A, Musaya J. *Schistosoma mattheei* and zoonotic schistosomiasis. Trends Parasitol. 2025;41(2):87–90. doi: 10.1016/j.pt.2024.12.008 39765449

[9] Laidemitt MR, Zawadzki ET, Brant SV, Mutuku MW, Mkoji GM, Loker ES. Loads of trematodes: discovering hidden diversity of paramphistomoids in Kenyan ruminants. Parasitology. 2017;144(2):131–47. doi: 10.1017/S0031182016001827 27762185 PMC5300004

[10] Pfukenyi DM, Mukaratirwa S. Amphistome infections in domestic and wild ruminants in East and Southern Africa: A review. Onderstepoort J Vet Res. 2018;85(1):e1–13. doi: 10.4102/ojvr.v85i1.1584 30456960 PMC6244199

[11] Borlase A, Rudge JW, Léger E, Diouf ND, Fall CB, Diop SD, et al. Spillover, hybridization, and persistence in schistosome transmission dynamics at the human-animal interface. Proc Natl Acad Sci U S A. 2021;118(41):e2110711118. doi: 10.1073/pnas.2110711118 34615712 PMC8521685

[12] Kayuni S, Cunningham L, Mainga B, Kumwenda D, Jnr DL, Chammudzi P, et al. Detection of male genital schistosomiasis (MGS) associated with human, zoonotic and hybrid schistosomes in Southern Malawi. BMC Infect Dis. 2024;24(1):839. doi: 10.1186/s12879-024-09732-z 39160482 PMC11331596

[13] Berrie AD. Snail problems in African schistosomiasis. Adv Parasitol. 1970;8:43–96. doi: 10.1016/s0065-308x(08)60251-1 4997516

[14] Wright CA. *Bulinus* on Aldabra and the subfamily Bulininae in the Indian Ocean area. Phil Trans R Soc Lond B. 1971;260(836):299–313. doi: 10.1098/rstb.1971.0016

[15] Mandahl-barth G. Intermediate hosts of schistosoma: African Biomphalaria and *Bulinus*. II. Bull World Health Organ. 1957;17(1):1–65. 13479773 PMC2537581

[16] Kane RA, Stothard JR, Emery AM, Rollinson D. Molecular characterization of freshwater snails in the genus *Bulinus*: a role for barcodes?. Parasit Vectors. 2008;1(1):15. doi: 10.1186/1756-3305-1-15 18544153 PMC2441610

[17] Zein-Eddine R, Djuikwo-Teukeng FF, Al-Jawhari M, Senghor B, Huyse T, Dreyfuss G. Phylogeny of seven *Bulinus* species originating from endemic areas in three African countries, in relation to the human blood fluke *Schistosoma haematobium*. BMC Evol Biol. 2014;14(1). doi: 10.1186/s12862-014-0271-3 PMC4295282 25528261

[18] Allan F, Sousa-Figueiredo JC, Emery AM, Paulo R, Mirante C, Sebastião A, et al. Mapping freshwater snails in north-western Angola: distribution, identity and molecular diversity of medically important taxa. Parasit Vectors. 2017;10(1):460. doi: 10.1186/s13071-017-2395-y 29017583 PMC5634851

[19] Tumwebaze I, Clewing C, Dusabe MC, Tumusiime J, Kagoro-Rugunda G, Hammoud C, et al. Molecular identification of *Bulinus* spp. intermediate host snails of Schistosoma spp. in crater lakes of western Uganda with implications for the transmission of the *Schistosoma haematobium* group parasites. Parasit Vectors. 2019;12(1):565. doi: 10.1186/s13071-019-3811-2 31775865 PMC6882369

[20] Chibwana FD, Tumwebaze I, Mahulu A, Sands AF, Albrecht C. Assessing the diversity and distribution of potential intermediate hosts snails for urogenital schistosomiasis: *Bulinus* spp. (Gastropoda: Planorbidae) of Lake Victoria. Parasit Vectors. 2020;13(1):418. doi: 10.1186/s13071-020-04281-1 32795373 PMC7427762

[21] Agola EL, Mwangi IN, Maina GM, Kinuthia JM, Mutuku MW. Transmission sites for *Schistosoma haematobium* and Schistosoma bovis identified in localities within the Athi River basin of Kenya using a PCR-RFLP assay. Heliyon. 2021;7(2):e06114. doi: 10.1016/j.heliyon.2021.e06114 33644442 PMC7889825

[22] Pennance T, Ame SM, Amour AK, Suleiman KR, Muhsin MA, Kabole F, et al. Transmission and diversity of *Schistosoma haematobium* and S. bovis and their freshwater intermediate snail hosts *Bulinus* globosus and B. nasutus in the Zanzibar Archipelago, United Republic of Tanzania. PLoS Negl Trop Dis. 2022;16(7):e0010585. doi: 10.1371/journal.pntd.0010585 35788199 PMC9286283

[23] Babbitt CR, Laidemitt MR, Mutuku MW, Oraro PO, Brant SV, Mkoji GM, et al. *Bulinus* snails in the Lake Victoria Basin in Kenya: Systematics and their role as hosts for schistosomes. PLoS Negl Trop Dis. 2023;17(2):e0010752. doi: 10.1371/journal.pntd.0010752 36763676 PMC9949660

[24] Tantrawatpan C, Vaisusuk K, Tanga CM, Pilap W, Bunchom N, Andrews RH, et al. Nuclear Intron Sequence Variation of the *Bulinus* globosus Complex (Mollusca: Planorbidae): Implications for Molecular Systematic Analyses. Biology (Basel). 2025;14(1):53. doi: 10.3390/biology14010053 39857284 PMC11761897

[25] Young ND, Stroehlein AJ, Wang T, Korhonen PK, Mentink-Kane M, Stothard JR, et al. Nuclear genome of *Bulinus* truncatus, an intermediate host of the carcinogenic human blood fluke *Schistosoma haematobium*. Nat Commun. 2022;13(1):977. doi: 10.1038/s41467-022-28634-9 35190553 PMC8861042

[26] Zhang S-M, Bu L, Lu L, Babbitt C, Adema CM, Loker ES. Comparative mitogenomics of freshwater snails of the genus *Bulinus*, obligatory vectors of *Schistosoma haematobium*, causative agent of human urogenital schistosomiasis. Sci Rep. 2022;12(1):5357. doi: 10.1038/s41598-022-09305-7 35354876 PMC8967911

[27] Bu L, Habib MR, Lu L, Mutuku MW, Loker ES, Zhang S-M. Transcriptional profiling of *Bulinus* globosus provides insights into immune gene families in snails supporting the transmission of urogenital schistosomiasis. Dev Comp Immunol. 2024;154:105150. doi: 10.1016/j.dci.2024.105150 38367887 PMC10932938

[28] Habib MR, Posavi M, Lekired A, Zhang S-M. Exploring the genome-wide transcriptomic responses of *Bulinus* truncatus to *Schistosoma haematobium* infection: An important host-parasite system involved in the transmission of human urogenital schistosomiasis. Mol Immunol. 2024;175:74–88. doi: 10.1016/j.molimm.2024.09.006 39307031 PMC12019995

[29] Bu L, Lu L, Laidemitt MR, Zhang S-M, Mutuku M, Mkoji G, et al. A genome sequence for Biomphalaria pfeifferi, the major vector snail for the human-infecting parasite *Schistosoma mansoni*. PLoS Negl Trop Dis. 2023;17(3):e0011208. doi: 10.1371/journal.pntd.0011208 36961841 PMC10075465

[30] Doležel J, Sgorbati S, Lucretti S. Comparison of three DNA fluorochromes for flow cytometric estimation of nuclear DNA content in plants. Physiologia Plantarum. 1992;85(4):625–31. doi: 10.1111/j.1399-3054.1992.tb04764.x

[31] Bu L, Zhong D, Lu L, Loker ES, Yan G, Zhang S-M. Compatibility between snails and schistosomes: insights from new genetic resources, comparative genomics, and genetic mapping. Commun Biol. 2022;5(1):940. doi: 10.1038/s42003-022-03844-5 36085314 PMC9463173

[32] Zhang S-M, Yan G, Lekired A, Zhong D. Genomic basis of schistosome resistance in a molluscan vector of human schistosomiasis. iScience. 2024;28(1):111520. doi: 10.1016/j.isci.2024.111520 39758819 PMC11699755

[33] Bolger AM, Lohse M, Usadel B. Trimmomatic: a flexible trimmer for Illumina sequence data. Bioinformatics. 2014;30(15):2114–20. doi: 10.1093/bioinformatics/btu170 24695404 PMC4103590

[34] Hahn C, Bachmann L, Chevreux B. Reconstructing mitochondrial genomes directly from genomic next-generation sequencing reads--a baiting and iterative mapping approach. Nucleic Acids Res. 2013;41(13):e129. doi: 10.1093/nar/gkt371 23661685 PMC3711436

[35] Dierckxsens N, Mardulyn P, Smits G. NOVOPlasty: de novo assembly of organelle genomes from whole genome data. Nucleic Acids Res. 2017;45(4):e18. doi: 10.1093/nar/gkw955 28204566 PMC5389512

[36] Jin J-J, Yu W-B, Yang J-B, Song Y, dePamphilis CW, Yi T-S, et al. GetOrganelle: a fast and versatile toolkit for accurate de novo assembly of organelle genomes. Genome Biol. 2020;21(1):241. doi: 10.1186/s13059-020-02154-5 32912315 PMC7488116

[37] Prjibelski A, Antipov D, Meleshko D, Lapidus A, Korobeynikov A. Using SPAdes De Novo Assembler. Curr Protoc Bioinformatics. 2020;70(1):e102. doi: 10.1002/cpbi.102 32559359

[38] Uliano-Silva M, Ferreira JGRN, Krasheninnikova K, Darwin Tree of Life Consortium, Formenti G, Abueg L, et al. MitoHiFi: a python pipeline for mitochondrial genome assembly from PacBio high fidelity reads. BMC Bioinformatics. 2023;24(1):288. doi: 10.1186/s12859-023-05385-y 37464285 PMC10354987

[39] Li H. Minimap2: pairwise alignment for nucleotide sequences. Bioinformatics. 2018;34(18):3094–100. doi: 10.1093/bioinformatics/bty191 29750242 PMC6137996

[40] Cheng H, Concepcion GT, Feng X, Zhang H, Li H. Haplotype-resolved de novo assembly using phased assembly graphs with hifiasm. Nat Methods. 2021;18(2):170–5. doi: 10.1038/s41592-020-01056-5 33526886 PMC7961889

[41] Donath A, Jühling F, Al-Arab M, Bernhart SH, Reinhardt F, Stadler PF, et al. Improved annotation of protein-coding genes boundaries in metazoan mitochondrial genomes. Nucleic Acids Res. 2019;47(20):10543–52. doi: 10.1093/nar/gkz833 31584075 PMC6847864

[42] Galaxy Community. The Galaxy platform for accessible, reproducible, and collaborative data analyses: 2024 update. Nucleic Acids Res. 2024;52(W1):W83–94. doi: 10.1093/nar/gkae410 38769056 PMC11223835

[43] Fourdrilis S, de Frias Martins AM, Backeljau T. Relation between mitochondrial DNA hyperdiversity, mutation rate and mitochondrial genome evolution in Melarhaphe neritoides (Gastropoda: Littorinidae) and other Caenogastropoda. Sci Rep. 2018;8(1):17964. doi: 10.1038/s41598-018-36428-7 30568252 PMC6299273

[44] Ghiselli F, Gomes-Dos-Santos A, Adema CM, Lopes-Lima M, Sharbrough J, Boore JL. Molluscan mitochondrial genomes break the rules. Philos Trans R Soc Lond B Biol Sci. 2021;376(1825):20200159. doi: 10.1098/rstb.2020.0159 33813887 PMC8059616

[45] Edgar RC. MUSCLE: multiple sequence alignment with high accuracy and high throughput. Nucleic Acids Res. 2004;32(5):1792–7. doi: 10.1093/nar/gkh340 15034147 PMC390337

[46] Kumar S, Stecher G, Li M, Knyaz C, Tamura K. MEGA X: Molecular Evolutionary Genetics Analysis across Computing Platforms. Mol Biol Evol. 2018;35(6):1547–9. doi: 10.1093/molbev/msy096 29722887 PMC5967553

[47] Folmer O, Black M, Hoeh W, Lutz R, Vrijenhoek R. DNA primers for amplification of mitochondrial cytochrome c oxidase subunit I from diverse metazoan invertebrates. Mol Mar Biol Biotechnol. 1994;3(5):294–9. 7881515

[48] Tamura K. Estimation of the number of nucleotide substitutions when there are strong transition-transversion and G+C-content biases. Mol Biol Evol. 1992;9(4):678–87. doi: 10.1093/oxfordjournals.molbev.a040752 1630306

[49] Tavaré S. Some probabilistic and statistical problems on the analysis of DNA sequences. Lectures in Mathematics of the Life Sciences. 1986;57–86.

[50] Felsenstein J. Confidence limits on phylogenies: an approach using the bootstrap. Evolution. 1985;39(4):783–91. doi: 10.1111/j.1558-5646.1985.tb00420.x 28561359

[51] Wickham H, Averick M, Bryan J, Chang W, McGowan L, François R, et al. Welcome to the Tidyverse. JOSS. 2019;4(43):1686. doi: 10.21105/joss.01686

[52] Lê S, Josse J, Husson F. FactoMineR: AnRPackage for Multivariate Analysis. J Stat Soft. 2008;25(1). doi: 10.18637/jss.v025.i01

[53] Wickham H. ggplot2. Springer International Publishing. 2016. doi: 10.1007/978-3-319-24277-4

[54] Neuwirth E. RColorBrewer: ColorBrewer palettes. 2022.

[55] Patterson CM, Burch JB. Chromosomes of pulmonate mollusks. In: Fretter V, Peake J. Pulmonates, Vol. 2A, Systematics, Evolution and Ecology. London, New York, and San Francisco: Academic Press. 1988;171–217.

[56] Goldman MA, LoVerde PT, Chrisman CL, Frankin DA. Chromosomal evolution in planorbid snails of the genera *Bulinus* and Biomphalaria. Malacologia. 1984;25(2):427–46.

[57] Adema CM, Hillier LW, Jones CS, Loker ES, Knight M, Minx P, et al. Whole genome analysis of a schistosomiasis-transmitting freshwater snail. Nat Commun. 2017;8:15451. doi: 10.1038/ncomms15451 28508897 PMC5440852

[58] Pennance T, Calvelo J, Tennessen JA, Burd R, Cayton J, Bollmann SR, et al. The genome and transcriptome of the snail Biomphalaria sudanica s.l.: immune gene diversification and highly polymorphic genomic regions in an important African vector of *Schistosoma mansoni*. BMC Genomics. 2024;25(1):192. doi: 10.1186/s12864-024-10103-w 38373909 PMC10875847

[59] Zhong D, Bu L, Habib MR, Lu L, Yan G, Zhang S-M. A haplotype-like, chromosome-level assembled and annotated genome of Biomphalaria glabrata, an important intermediate host of schistosomiasis and the best studied model of schistosomiasis vector snails. PLoS Negl Trop Dis. 2024;18(2):e0011983. doi: 10.1371/journal.pntd.0011983 38421953 PMC10903818

[60] Bouchet P, Rocroi JP, Hausdorf B, Kaim A, Kano Y, Nützel A, et al. Revised classification, nomenclator and typification of gastropod and monoplacophoran families. Malacologia. 2017;61(1-2):1–526. 10.4002/040.061.0201

[61] nam P, Tripathi NK, Kour P. Karyotypic and Morphometric Studies in Two Species of Family Planorbidae (Gastropoda: Mollusca). IntJCurrMicrobiolAppSci. 2018;7(09):1180–7. doi: 10.20546/ijcmas.2018.709.140

[62] Natarajan R, Burch J, Gismann A. Cytological studies of Planorbidae. 2 Some African Planorbidae, Bulininae and Planorbininae. Malacologia. 1965;2:239–51.

[63] Burch JB. Chromosomes of intermediate hoss of human bilharziasis. Malacologia. 1967;5(2):127–35.

[64] Claugher D. Karyotype analysis of bulinid snails. Bull World Health Organ. 1971;45(6):855–8. 5317019 PMC2427983

[65] Brown DS, Wright CA. On a polyploid complex of freshwater snails (Planorbidae: *Bulinus*) in Ethiopia. Journal of Zoology. 1972;167(1):97–132. doi: 10.1111/j.1469-7998.1972.tb01723.x

[66] Goldman MA, LoVerde PT, Chrisman CL. Comparative karyology of the freshwater snails *Bulinus* tropicus and B. natalensis. Can J Genet Cytol. 1980;22(3):361–7. doi: 10.1139/g80-044 7448622

[67] Goldman MA, LoVerde PT, Chrisman CL. Hybrid origin of polyploidy in freshwater snails of the genus *bulinus* (mollusca: planorbidae). Evolution. 1983;37(3):592–600. doi: 10.1111/j.1558-5646.1983.tb05576.x 28563304

[68] McIntyre PJ. Cytogeography and genome size variation in the Claytonia perfoliata (Portulacaceae) polyploid complex. Ann Bot. 2012;110(6):1195–203. doi: 10.1093/aob/mcs187 22962302 PMC3478050

[69] Rothleutner JJ, Friddle MW, Contreras RN. Ploidy Levels, Relative Genome Sizes, and Base Pair Composition in Cotoneaster. J Amer Soc Hort Sci. 2016;141(5):457–66. doi: 10.21273/jashs03776-16

[70] Brown DS. A tetraploid freshwater snail (Planorbidae: *Bulinus*) in the highlands of Kenya. Journal of Natural History. 1976;10(3):257–67. doi: 10.1080/00222937600770191

[71] Burch JB, Bruce JI, Rudolph PH, Mallett JC, Banhawy MA. The chromosome numbers of four populations of bulinine snails from Lake Nasser, Egypt. Tropenmed Parasitol. 1979;30(2):174–8. 483381

[72] Yaseen AE. Cytogenetics and Biology of the Intermediate Host of Human Bilharziasis, *Bulinus* truncatus Common in Upper Egypt. cytologia. 1993;58(1):53–60. doi: 10.1508/cytologia.58.53

[73] DeJong RJ, Morgan JA, Paraense WL, Pointier JP, Amarista M, Ayeh-Kumi PF, et al. Evolutionary relationships and biogeography of Biomphalaria (Gastropoda: Planorbidae) with implications regarding its role as host of the human bloodfluke, *Schistosoma mansoni*. Mol Biol Evol. 2001;18(12):2225–39. doi: 10.1093/oxfordjournals.molbev.a003769 11719572

[74] Gregory TR, Nicol JA, Tamm H, Kullman B, Kullman K, Leitch IJ, et al. Eukaryotic genome size databases. Nucleic Acids Res. 2007;35(Database issue):D332-8. doi: 10.1093/nar/gkl828 17090588 PMC1669731

[75] Jeffery NW, Hultgren K, Chak STC, Gregory TR, Rubenstein DR. Patterns of genome size variation in snapping shrimp. Genome. 2016;59(6):393–402. doi: 10.1139/gen-2015-0206 27171678

[76] Moraes IRR, Pardo LM, Araya-Jaime C, Wolf MR, Yasui GS, Solano-Iguaran JJ, et al. Patterns of genome size variation in caridean shrimps: new estimates for non-gambarelloides Synalpheus species. Genome. 2022;65(8):459–68. doi: 10.1139/gen-2022-0015 35917258

[77] Alvarez-Fuster A, Juan C, Petitpierre E. Genome size in Tribolium flour-beetles: inter- and intraspecific variation. Genet Res. 1991;58(1):1–5. doi: 10.1017/s0016672300029542

[78] Ellis LL, Huang W, Quinn AM, Ahuja A, Alfrejd B, Gomez FE, et al. Intrapopulation genome size variation in D. melanogaster reflects life history variation and plasticity. PLoS Genet. 2014;10(7):e1004522. doi: 10.1371/journal.pgen.1004522 25057905 PMC4109859

[79] Blommaert J, Riss S, Hecox-Lea B, Mark Welch DB, Stelzer CP. Small, but surprisingly repetitive genomes: transposon expansion and not polyploidy has driven a doubling in genome size in a metazoan species complex. BMC Genomics. 2019;20(1):466. doi: 10.1186/s12864-019-5859-y 31174483 PMC6555955

[80] Neiman M, Paczesniak D, Soper DM, Baldwin AT, Hehman G. Wide variation in ploidy level and genome size in a New Zealand freshwater snail with coexisting sexual and asexual lineages. Evolution. 2011;65(11):3202–16. doi: 10.1111/j.1558-5646.2011.01360.x 22023586

[81] Larkin K, Tucci C, Neiman M. Effects of polyploidy and reproductive mode on life history trait expression. Ecol Evol. 2016;6(3):765–78. doi: 10.1002/ece3.1934 26865964 PMC4739562

[82] McElroy KE, Müller S, Lamatsch DK, Bankers L, Fields PD, Jalinsky JR, et al. Asexuality Associated with Marked Genomic Expansion of Tandemly Repeated rRNA and Histone Genes. Mol Biol Evol. 2021;38(9):3581–92. doi: 10.1093/molbev/msab121 33885820 PMC8382920

[83] Osnas EE, Lively CM. Host ploidy, parasitism and immune defence in a coevolutionary snail-trematode system. J Evol Biol. 2006;19(1):42–8. doi: 10.1111/j.1420-9101.2005.00994.x 16405575

[84] King KC, Seppälä O, Neiman M. Is more better? Polyploidy and parasite resistance. Biol Lett. 2012;8(4):598–600. doi: 10.1098/rsbl.2011.1152 22258448 PMC3391438

[85] Hagen ER, Mason CM. Differences in pathogen resistance between diploid and polyploid plants: a systematic review and meta‐analysis. Oikos. 2023;2024(5). doi: 10.1111/oik.09908

[86] Adams KL. Evolution of duplicate gene expression in polyploid and hybrid plants. J Hered. 2007;98(2):136–41. doi: 10.1093/jhered/esl061 17208934

[87] Chen ZJ. Genetic and epigenetic mechanisms for gene expression and phenotypic variation in plant polyploids. Annu Rev Plant Biol. 2007;58:377–406. doi: 10.1146/annurev.arplant.58.032806.103835 17280525 PMC1949485

[88] Yoo M-J, Liu X, Pires JC, Soltis PS, Soltis DE. Nonadditive gene expression in polyploids. Annu Rev Genet. 2014;48:485–517. doi: 10.1146/annurev-genet-120213-092159 25421600

[89] Wang L, Jia G, Jiang X, Cao S, Chen ZJ, Song Q. Altered chromatin architecture and gene expression during polyploidization and domestication of soybean. Plant Cell. 2021;33(5):1430–46. doi: 10.1093/plcell/koab081 33730165 PMC8254482

[90] Rollinson D, Stothard JR, Southgate VR. Interactions between intermediate snail hosts of the genus *Bulinus* and schistosomes of the *Schistosoma haematobium* group. Parasitology. 2001;123 Suppl:S245-60. doi: 10.1017/s0031182001008046 11769287

[91] Li J-T, Wang Q, Huang Yang M-D, Li Q-S, Cui M-S, Dong Z-J, et al. Parallel subgenome structure and divergent expression evolution of allo-tetraploid common carp and goldfish. Nat Genet. 2021;53(10):1493–503. doi: 10.1038/s41588-021-00933-9 34594040 PMC8492472

[92] Kuhl H, Du K, Schartl M, Kalous L, Stöck M, Lamatsch DK. Author Correction: Equilibrated evolution of the mixed auto-/allopolyploid haplotype-resolved genome of the invasive hexaploid Prussian carp. Nat Commun. 2022;13(1):4638. doi: 10.1038/s41467-022-32470-2 35941146 PMC9359990

[93] Liu S, Li K, Dai X, Qin G, Lu D, Gao Z, et al. A telomere-to-telomere genome assembly coupled with multi-omic data provides insights into the evolution of hexaploid bread wheat. Nat Genet. 2025;57(4):1008–20. doi: 10.1038/s41588-025-02137-x 40195562 PMC11985340

[94] Zan Y, Chen S, Ren M, Liu G, Liu Y, Han Y, et al. The genome and GeneBank genomics of allotetraploid Nicotiana tabacum provide insights into genome evolution and complex trait regulation. Nat Genet. 2025;57(4):986–96. doi: 10.1038/s41588-025-02126-0 40140587 PMC11985347

[95] Zhang S-M, Bu L, Laidemitt MR, Lu L, Mutuku MW, Mkoji GM, et al. Complete mitochondrial and rDNA complex sequences of important vector species of Biomphalaria, obligatory hosts of the human-infecting blood fluke, *Schistosoma mansoni*. Sci Rep. 2018;8(1):7341. doi: 10.1038/s41598-018-25463-z 29743617 PMC5943310

[96] Stothard JR, Hughes S, Rollinson D. Variation within the internal transcribed spacer (ITS) of ribosomal DNA genes of intermediate snail hosts within the genus *Bulinus* (Gastropoda: Planorbidae). Acta Trop. 1996;61(1):19–29. doi: 10.1016/0001-706x(95)00137-4 9133161

[97] Jørgensen A, Jørgensen LVG, Kristensen TK, Madsen H, Stothard JR. Molecular phylogenetic investigations of *Bulinus* (Gastropoda: Planorbidae) in Lake Malawi with comments on the topological incongruence between DNA loci. Zoologica Scripta. 2007;36(6):577–85. doi: 10.1111/j.1463-6409.2007.00298.x

[98] Jørgensen A, Madsen H, Nalugwa A, Nyakaana S, Rollinson D, Stothard JR, et al. Molecular phylogenetic analysis of *Bulinus* (Gastropoda: Planorbidae) with conserved nuclear genes. Zoologica Scripta. 2010;40:126–36.

[99] Nalugwa A, Jørgensen A, Nyakaana S, Kristensen TK. Molecular phylogeny of *Bulinus* (Gastropoda: Planorbidae) reveals the presence of three species complexes in the Albertine Rift freshwater bodies. Intl J Genet Mol Biol. 2010;2(7):130–9.

[100] Tumwebaze I, Clewing C, Chibwana FD, Kipyegon JK, Albrecht C. Evolution and Biogeography of Freshwater Snails of the Genus *Bulinus* (Gastropoda) in Afromontane Extreme Environments. Front Environ Sci. 2022;10. doi: 10.3389/fenvs.2022.902900

[101] Andrus PS, Joof E, Wade CM. Differentiation of *Bulinus senegalensis* and *Bulinus* forskalii Snails in West Africa Using Morphometric Analysis. Acta Parasitol. 2024;69(1):1016–26. doi: 10.1007/s11686-024-00830-1 38502474 PMC11001693

[102] Stothard JR, Llewellyn-Hughes J, Griffin CE, Hubbard SJ, Kristensen TK, Rollinson D. Identification of snails within the *Bulinus* africanus group from East Africa by multiplex SNaPshot trade mark analysis of single nucleotide polymorphisms within the cytochrome oxidase subunit I. Mem Inst Oswaldo Cruz. 2002;97 Suppl 1:31–6. doi: 10.1590/s0074-02762002000900008 12426591

[103] Bogart JP, Bi K. Genetic and genomic interactions of animals with different ploidy levels. Cytogenet Genome Res. 2013;140(2–4):117–36. doi: 10.1159/000351593 23751376

[104] Zhong J, Yi S, Ma L, Wang W. Evolution and phylogeography analysis of diploid and polyploid Misgurnus anguillicaudatus populations across China. Proc Biol Sci. 2019;286(1901):20190076. doi: 10.1098/rspb.2019.0076 31014220 PMC6501937

[105] Brown DS, Wright CA. A new species of*Bulinus*(Mollusca: Gastropoda) from temporary freshwater pools in Kenya. Journal of Natural History. 1978;12(2):217–29. doi: 10.1080/00222937800770091

[106] Jelnes JE. Experimental taxonomy of *Bulinus*. 3. Electrophoretic observations on B. forskalii, B. browni, B. barthi and B. scalaris from East Africa, with additional electrophoretic data on the subgenus *Bulinus* s. s from other part of Africa. Steenstrupia. 1980;6:177–93.

[107] Toews DPL, Brelsford A. The biogeography of mitochondrial and nuclear discordance in animals. Mol Ecol. 2012;21(16):3907–30. doi: 10.1111/j.1365-294X.2012.05664.x 22738314

[108] Paczesniak D, Jokela J, Larkin K, Neiman M. Discordance between nuclear and mitochondrial genomes in sexual and asexual lineages of the freshwater snail Potamopyrgus antipodarum. Mol Ecol. 2013;22(18):4695–710. doi: 10.1111/mec.12422 23957656

[109] Nolan JR, Bergthorsson U, Adema CM. Physella acuta: atypical mitochondrial gene order among panpulmonates (Gastropoda). J Molluscan Stud. 2014;80(4):388–99. doi: 10.1093/mollus/eyu025 25368439 PMC4214460

[110] David P, Degletagne C, Saclier N, Jennan A, Jarne P, Plénet S, et al. Extreme mitochondrial DNA divergence underlies genetic conflict over sex determination. Curr Biol. 2022;32(10):2325-2333.e6. doi: 10.1016/j.cub.2022.04.014 35483362

