# Supplementary figures and images for: Complexity of schistosome vector bulinine snails in Kenya: Insights from nuclear genome size variation, complete mitochondrial genome sequence, and morphometric analysis
Source: PLoS Negl Trop Dis. 2025 Jul 14;19(7):e0013305. doi: 10.1371/journal.pntd.0013305 (PMC12274006; doi:10.1371/journal.pntd.0013305)

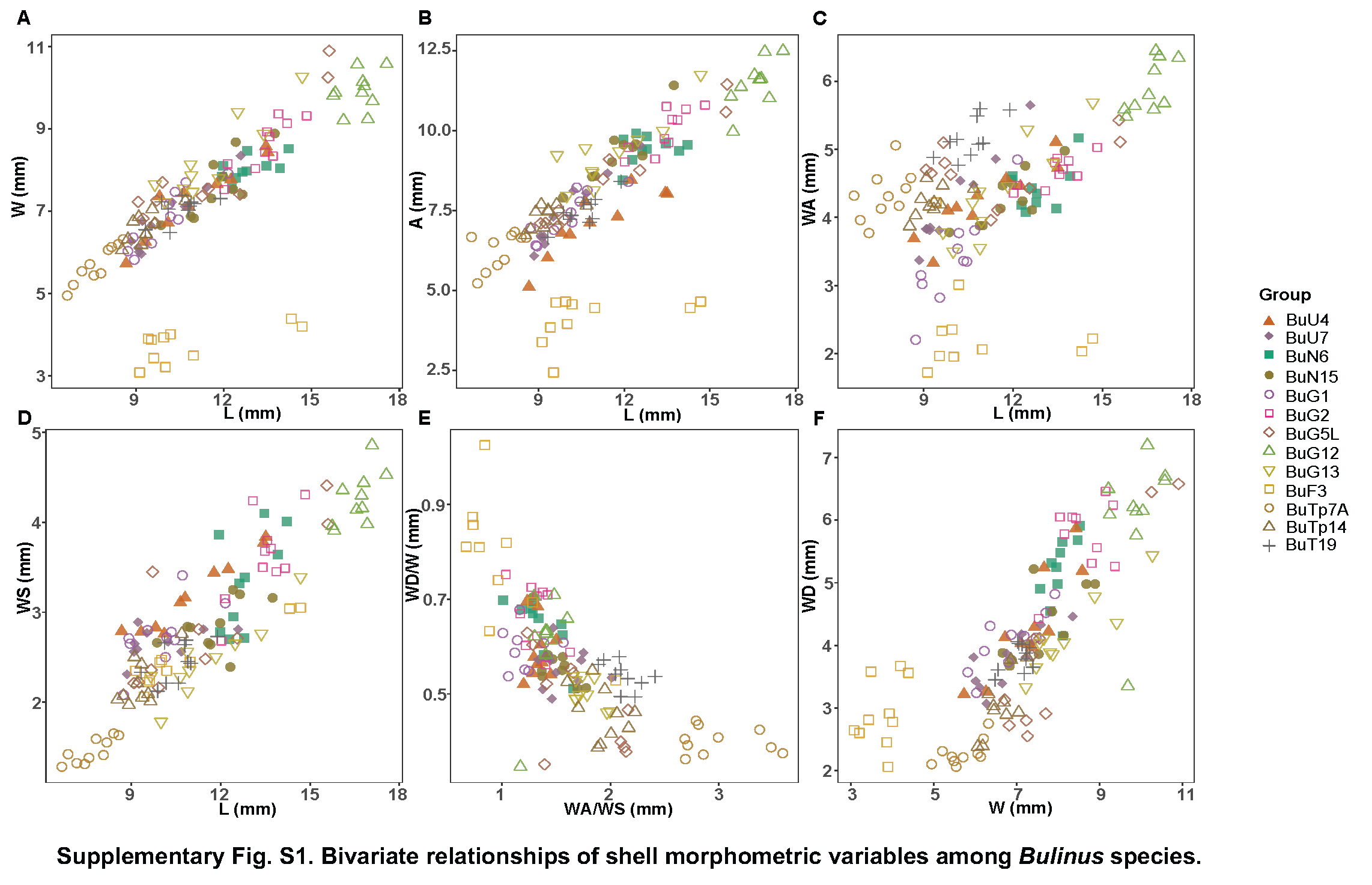

Supplement: S1 Fig — (TIFF) [file pntd.0013305.s005.tiff]

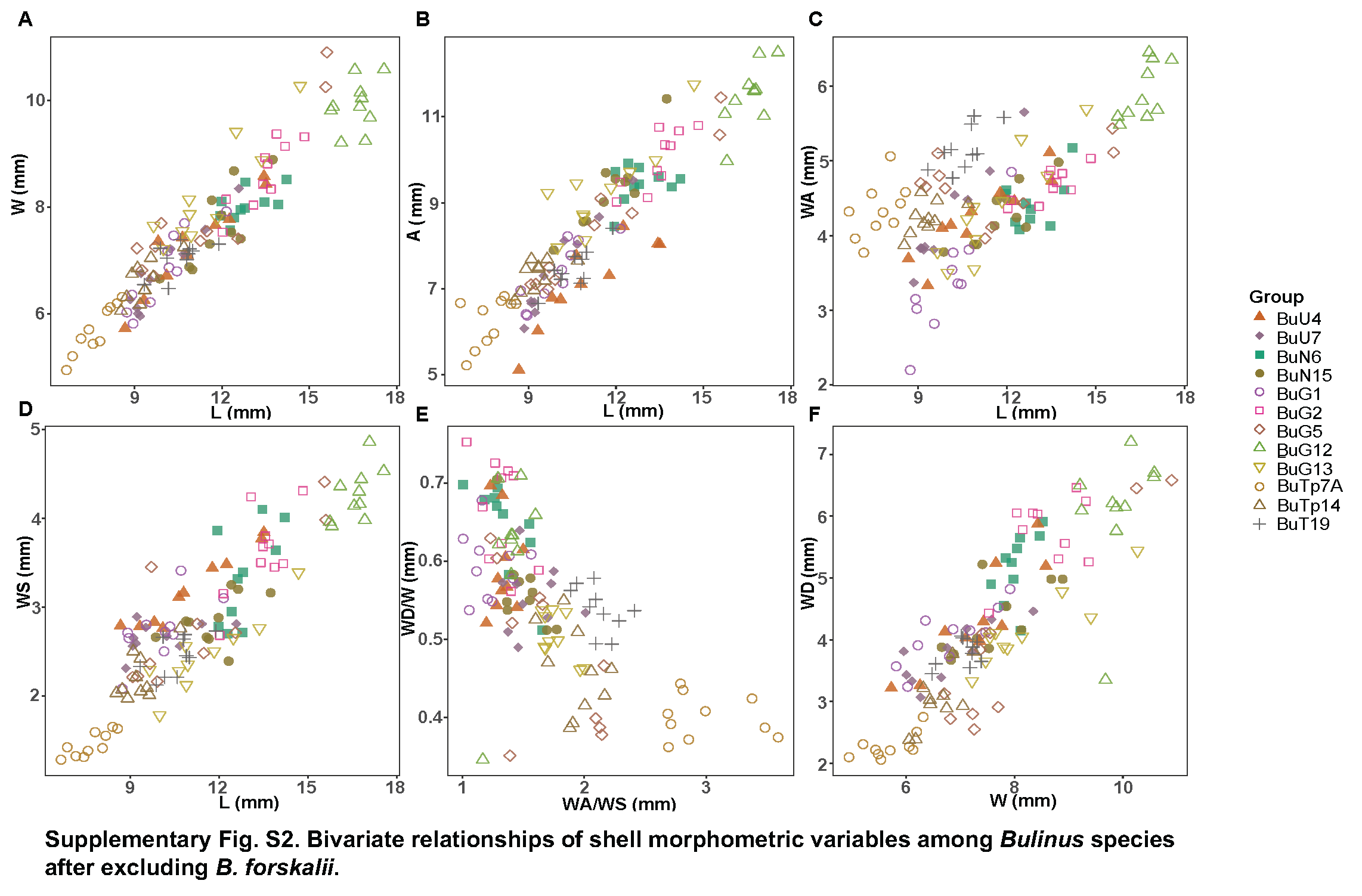

Supplement: S2 Fig — (TIFF) [file pntd.0013305.s006.tiff]
